# Extremely compact sources (ECS): a new potential field filtering method

**DOI:** 10.1038/s41598-024-62751-3

**Published:** 2024-05-25

**Authors:** Marco Maiolino, Giovanni Florio, Maurizio Fedi

**Affiliations:** Dipartimento di Scienze Della Terra Dell’Ambiente e Delle Risorse, University of Naples “Federico II”, Naples, Italy

**Keywords:** Potential field, Inversion, Filtering, Gravity, Applied Geophysics, Geomagnetism, Geophysics

## Abstract

We present a new filtering method for potential fields, based on modelling the fields in terms of very compact solutions, i.e., the sources are expected to occupy the smallest allowable volume in the source domain. The selected solutions, which we call “Extremely Compact Sources” (ECS) form a sort of atomized model, which still satisfies the non-unique inverse problem of gravity and magnetic fields. The ECS model is not only characterized by sparsity, but also by large values of the physical property (density or magnetic susceptibility). The sparse nature of the model allows for the definition of a highly localized filter, which can be obtained by simply specifying the atoms to be selected in a given area. This feature allows managing tasks normally impossible with traditional filters, such as the separation of interfering anomalies having a similar wavenumber content. In addition, the procedure can perform a very effective regional/residual separation. We demonstrate the method on synthetic cases and apply it in the real case of gravity data of Campi Flegrei volcanic area (Italy), where we use the ECS filtering to isolate the gravity effect of the Mount Olibano dome.

## Introduction

Potential field anomalies are generated by lateral contrasts of density/magnetization within the Earth. Often anomalies from more sources interfere with each other. Thus, it may be difficult to separate the anomaly generated by the source of interest from other local and regional fields. The interference effect is particularly subtle, yet severe, when the sources are at roughly the same depth. In this case, the shape and amplitude of the individual anomalies is strongly altered, and their identification, separation and modelling become very difficult to achieve. Some efforts have been made to address the problem of separating anomalies that overlap and interfere at similar wavenumber ranges. Fedi and Quarta^1^ tried to operate this kind of local filtering with the discrete wavelet transform. Zhu et al.^2^ showed the relation between the so-called trajectory matrix of the potential field and the geological model, demonstrating how the first few greatest singular values of the trajectory matrix of a potential field are related to the regional field data. Then, they established a low-rank matrix decomposition model in order to operate the separation. In this context, we introduce a new strategy for solving local interferences of anomalies, which we call Extremely Compact Sources (ECS) filtering. Decomposing the field in terms of extremely compact sources is similar to the atomization decomposition in the wavelet domain, performed by Mallat and Zhang^3^ with the matching pursuit algorithm (e.g.^4^). By this method, Mallat and Zhang decomposed the signal over a family of functions (time–frequency “atoms”) that are well localized in both space and wavenumber domains. We will use the same idea of “atom” in the space domain, by selecting an inverse model in which the different sources reach the smallest possible volume both in vertical and horizontal directions, i.e. an ECS, or atomized, model. Due to their reduced source volume, the atomized sources will have unrealistically high densities in the gravity case or magnetizations in the magnetic case. Due to the non-uniqueness of the inverse problem of potential fields, this “atomized” model is one of the infinite sets of different source distributions satisfying the inverse problem, meaning that they can equally well fit the data. Obviously, this model is not intended for interpretation, it represents just an equivalent source model. Our goal is only to exploit the extreme concentration of the sources to easily manage them by setting up an original filtering strategy. In the following Sections, we will describe the Extremely Compact Sources approach and will apply it to synthetic and real gravity data, showing its ability to simultaneously operate a local separation of interfering anomalies and a regional/residual separation.

## Methods

The key of ECS filtering is to invert a gravity (or magnetic) dataset for obtaining a model, ${\textbf{m}}_{\text{ecs}}$, in which the various sources are extremely compact and well separated (atoms). To obtain such a model, we use a type of linear inversion producing compact models, similar to those proposed by Last and Kubik^5^ or Portniaguine and Zhdanov^6^. We will first describe the algorithm for the compact inversion and then the filtering strategy, which will be applied to synthetic and real data.

### Source atomization

The potential field forward problem has the form:

1 $$\textbf{d}=\textbf{A}\textbf{m}$$

where $\textbf{A}$ is the gravity or magnetic sensitivity matrix, $\textbf{d}$ is the data vector and $\textbf{m}$ is the unknown vector of the model parameters. The inverse problem is an ill-posed problem, affected by numerical instability, which is usually addressed through the Tikhonov regularization^7^:

2 $$P^{\mu } \;\left( {\textbf{m}} \right)\mathop = \limits_{\phantom{0}} \arg \mathop {\min }\limits_{{\textbf{m}}} \left\{ {\left\Vert( {{\textbf{Am}} - {\textbf{d}}}\right)\Vert_{2}^{2} + \mu^{2} \Vert{\textbf{Lm}}\Vert_{2}^{2} } \right\}$$

where ${\Vert \textbf{A}\textbf{m}-\textbf{d}\Vert }_{2}$ is the L_2_ norm of the misfit between predicted $\left(\textbf{A}\textbf{m}\right)$ and observed data ($\textbf{d})$. The second term in Eq. (2) is the side constraint, in which $\textbf{L}$ is the regularization matrix, measuring some properties of the model, and $\mu$ is the regularization parameter, that weighs the minimization of the side constraint with respect to that of the residual norm.

Last and Kubik proposed a method aiming at maximizing the compactness of the source distribution. By subdividing the source volume in $M$ homogeneous blocks, the gravity effect at the ${i}^{th}$ data point is given by:

3 $${g}_{i}=\sum_{k=1}^{M}{a}_{ik}{m}_{k}+{e}_{i}\,\,\,\, i =1...N$$

where ${m}_{k}$ is density of the *k*th block, ${e}_{i}$ is the error associated with the ${i}_{th}$ data point, and ${a}_{ik}$ is the element of matrix $\textbf{A}$, representing the gravity effect of the *k*th block with unit-density at the ${i}_{th}$ measurement point. To maximize the compactness of the model, Last and Kubik proposed to minimize iteratively the area (or the volume in 3D) of the source using the following weighting function:

4 $${L}_{k}={{(m}_{k}^{2}+\varepsilon )}^{-1}$$

where $\varepsilon$ is a small positive number, introduced to avoid singularities.

Using Eq. (4), the objective function (Eq. 2) becomes:

5 $${P}^{\mu }\left(\textbf{m}\right)=\text{arg}\underset{\textbf{m}}{\text{min}}\left\{{\Vert \textbf{A}\textbf{m}-\textbf{d}\Vert }_{2}^{2}+{\mu }^{2}\sum_{k=1}^{M}\frac{{m}_{k}^{2}}{{m}_{k}^{2}+{\varepsilon }^{2}}\right\}.$$

We will here use a model-weighting function ${W}_{z}$, based on the depth weighting function. Such function, originally proposed by^8–10^, has its elements at a given depth *z* in the 3D model defined as:

6 $$w\left(z\right)=\frac{1}{{z}^{\beta /2}}$$

So, the weighting function (Eq. 4) becomes:

7 $${L}_{k}=\frac{{w}_{zk}}{{m}_{k}^{2}+{\varepsilon }^{2}}$$

We thus modify Eq. (5) as:

8 $${P}^{\mu }\left(\textbf{m}\right)=\text{arg }\underset{\textbf{m}}{\text{min}}\left\{\sum_{i=1}^{N}{\Vert \textbf{A}\textbf{m}-\textbf{d}\Vert }_{2}^{2}+{\mu }^{2}\sum_{k=1}^{M}\frac{{w}_{zk}{m}_{k}^{2}}{{m}_{k}^{2}+{\varepsilon }^{2} }\right\}$$

where ${w}_{zk}$ form the diagonal matrix ${\textbf{W}}_{z}$, whose elements are constant for each * z*_*th*_ layer. The inversion proceeds iteratively, with the weighting matrix updated at each iteration using the model obtained in the previous one. In this way, we may provide models with an increasing compactness, whose fields will fit the data equally well.

The key-concept of our filtering strategy is to choose, among all these models, the one having the sources with the smallest volumes (atoms). We emphasize that while these atoms are not intended to represent any real geological source, they are perfectly functional in separating the field into multiple components. This will be shown in the next section.

## ECS Filtering

**Figure 1:**
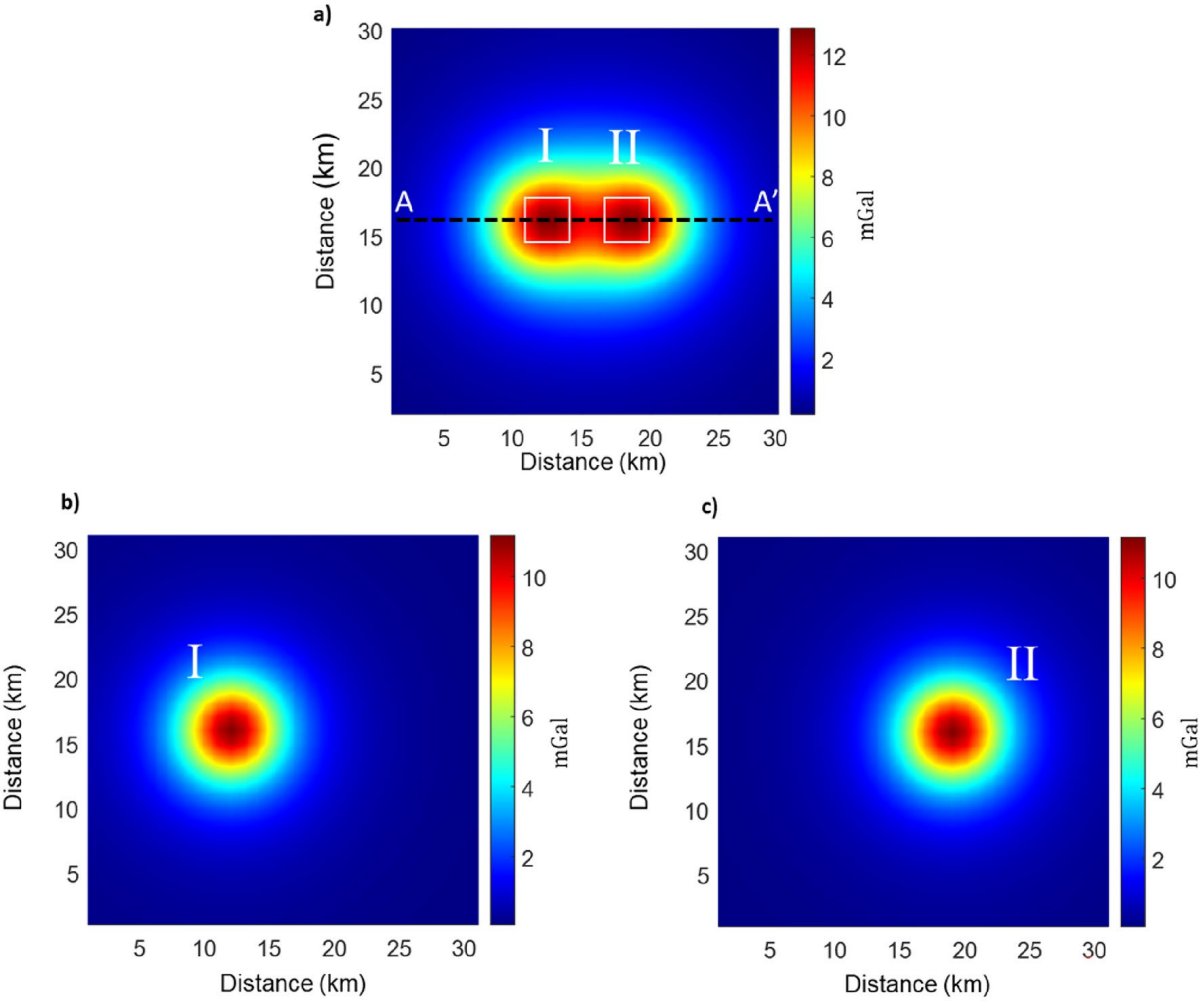
(**a**) Gravity of two 4 × 4 × 2 km^3^ prismatic sources, with a density contrast of 1000 kg/m^3^ with respect to the background, both with a depth to the top of 4 km. The dashed line marks the trace of the profile shown in Fig. 2; (**b**) Synthetic gravity field of the western source; (**c**) Synthetic gravity field of the eastern source.

To illustrate the filtering procedure, we present its application to a simple synthetic gravity anomaly generated by two identical 4 × 4 × 2 km^3^ prismatic sources, with a density contrast of 1000 $\text{kg}/{\text{m}}^{3}$ with respect to the background, both having their top at 4 km depth (Fig. 1a). The horizontal distance between the two sources is only 2 km, so their respective field components interfere severely.

**Figure 2:**
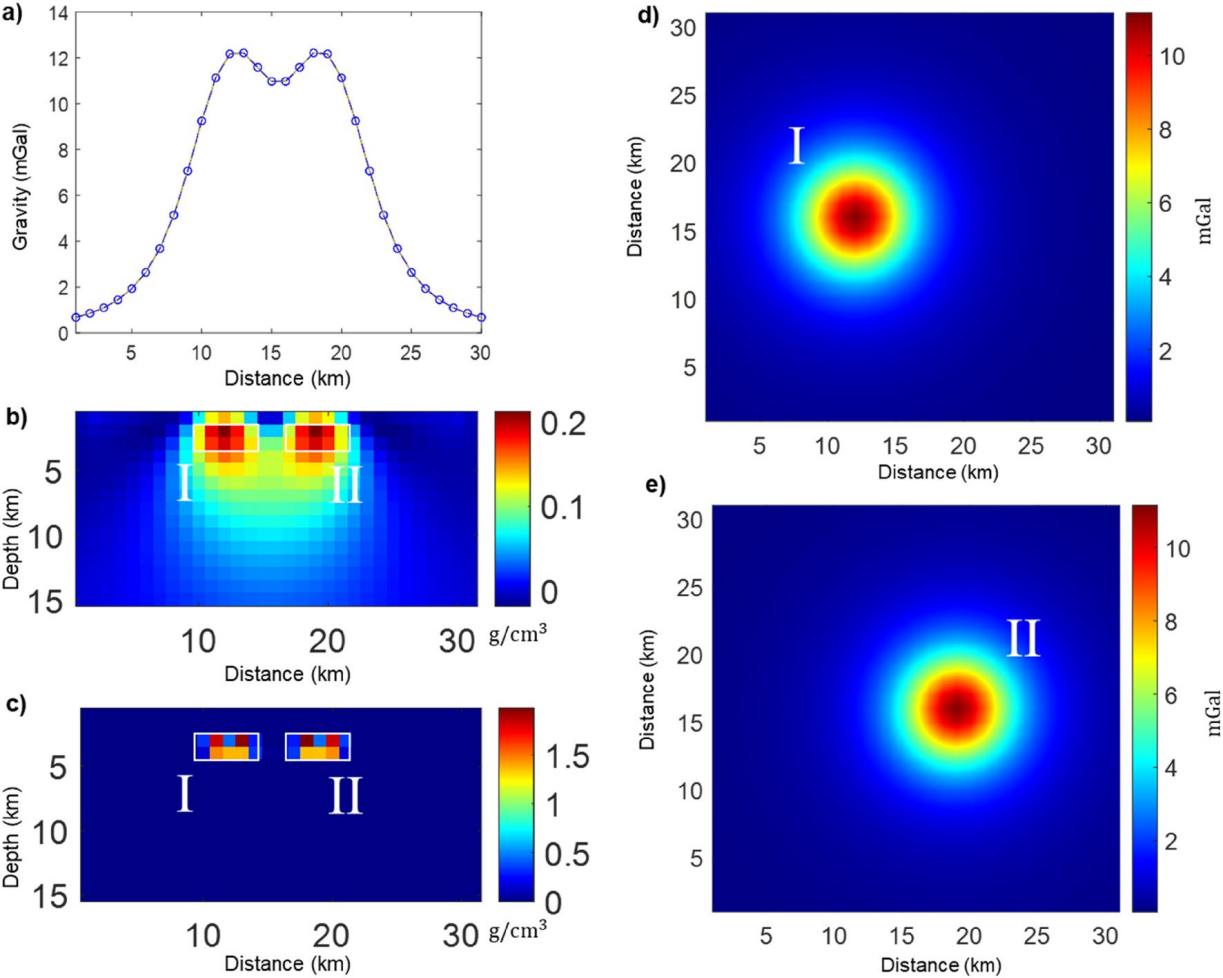
(**a**) Gravity field caused by the two prismatic sources shown in Fig. 1, along the profile A-A’ shown in Fig. 1a. (**b**) Vertical section of the obtained **m**_ECS_ at the 3rd iteration along the selected profile; (**c**) Vertical section of the obtained **m**_ECS_ at the 8th iteration along the selected profile. The symbols “I” and “II” identify the true prismatic sources, outlined by the white boxes. Note that the two models in (**b**) and (**c**) reproduce equally well the gravity field in (**a**). (**d**,**e**) Isolated gravity fields resulting from ECS filtering of the gravity data in Fig. 1a. (**d**) Gravity field of the western source; (**e**) Gravity field of the eastern source.

The gravity field of Fig. 1a is inverted iteratively according to Eq. (8). At the first iteration the maximum smoothness solution is obtained; then, with further iterations the sources volume progressively decreases, while the density increases. Thus, the model is characterized by highly concentrated and well separated blocks. Density of cells below some threshold, say ${10}^{-6}\text{g}/{\text{cm}}^{3}$, may be set to zero. Figure 2b,c show the different degree of source compaction at iterations 3 and 8. At the 3rd iteration (Fig. 2b) the model is not concentrated enough to isolate the single sources completely. Increasing the number of iterations allows a greater compactness up to obtain very small source volumes. This leads to a good source separation at the 8th iteration (Fig. 2c).

Having computed the ECS model of Fig. 2c, the filtering process is done by:

- selecting the area centered on the anomalies to study; if necessary, using vertical (integer or fractional) differentiation^11^, to obtain a gravity map with more resolution:
- defining the source volume corresponding to this area;
- exploring the source volume and identifying an ECS subset within it, ${\textbf{m}}_{\text{ECS}}$;
- Once the ECS atoms has been selected, the gravity field generated by them (representing of the local or regional component) can be computed using equation:
  9 $${\textbf{d}}_{\text{filt}}={\textbf{A}\textbf{m}}_{\text{ECS}}$$

In the case of Fig. 1, we select two models, **m**_I_ and **m**_II_, related to the two separated group of sources of Fig. 2c and computed the gravity fields of the two isolated sources by Eq. (9). To validate our procedure, we compare the true gravity field generated by each of the prismatic sources separately (Fig. 1b,c), with the isolated gravity fields resulting from ECS filtering (Fig. 2d,e).

**Figure 3:**
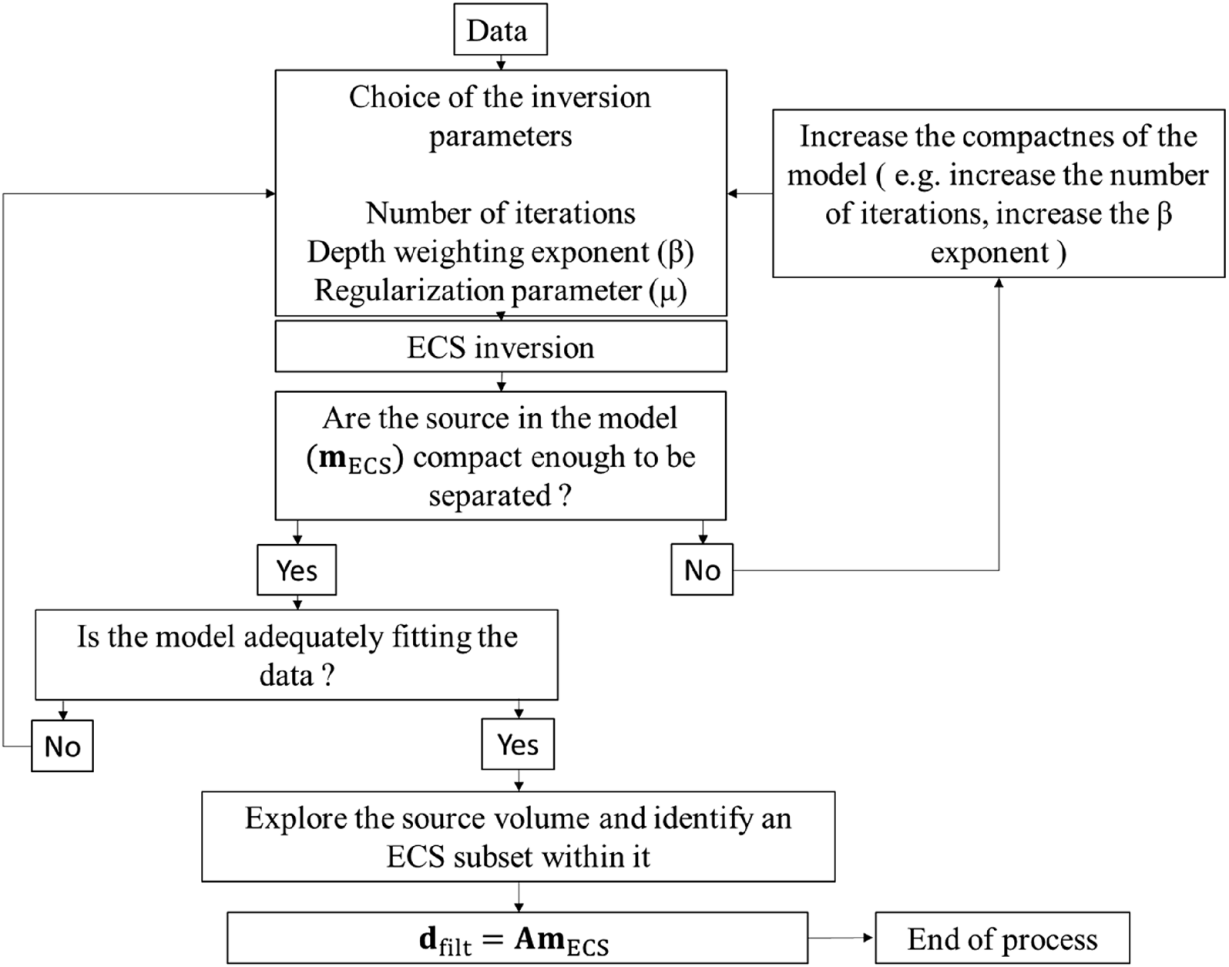
ECS filtering flow-chart.

The validity of our result is demonstrated by the relative residuals, expressed as the ratio of the norm of the difference between the observed and predicted data to the norm of the observed data. We found a relative error of $0.2759\%$ for the western source and $0.2725\%$ for the eastern source. The ECS filtering workflow is illustrated in Fig. 3.

### ECS filtering of local sources in presence of regional fields

**Figure 4:**
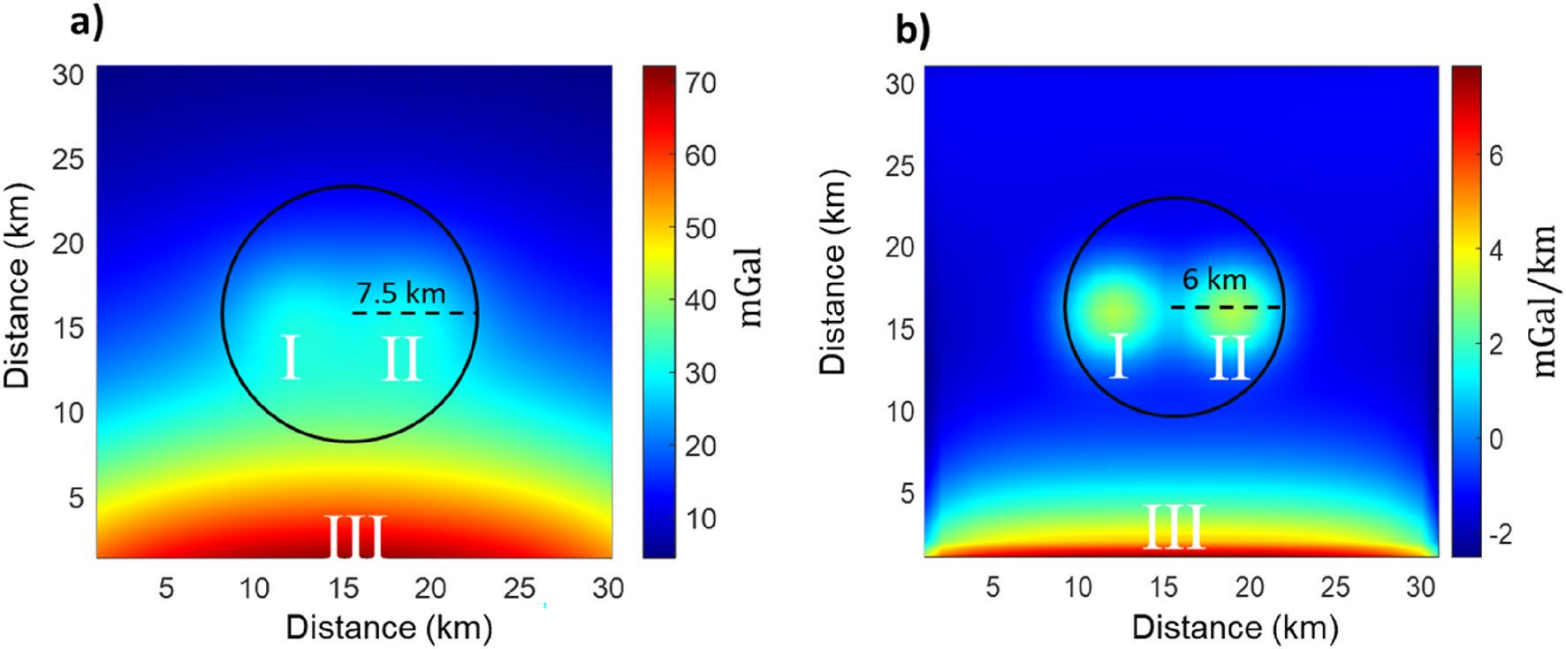
Synthetic gravity field and vertical derivative. (**a**) Synthetic gravity field. The local component consists in the same gravity field as in Fig. 1a (sources I and II). A regional trend decreasing from south to north and generated by a distant source (III) hides the real amplitude and shape of the local components. The black circle individuates the area in which we expect to find local ECS. (**b**) first order vertical derivative of the field in (**a**). A vertical differentiation can help a better individuation of the area in which ECS related to the selected anomalies could be found.

We added the contribution of a distant dense source to the gravity field in Fig. 1a, simulating the effect of a regional trend contaminating the field in the area of interest (Fig. 4). As already described, the first step in our filtering approach includes an analysis of the gravity anomaly aimed at defining an area in which we expect to find ECS representing the sources I and II. This is simply done by studying the shape of the anomalies.

**Figure 5:**
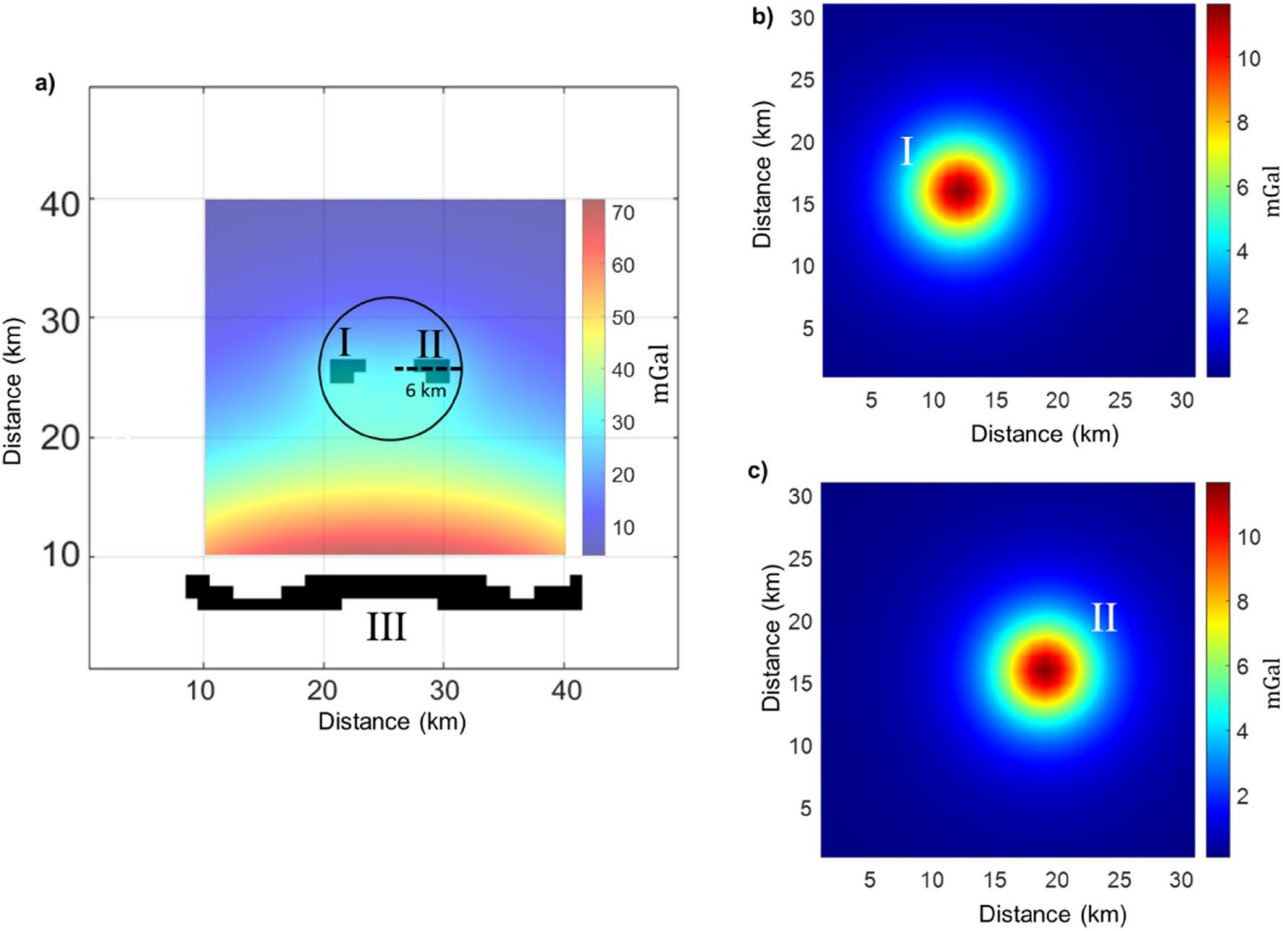
ECS model **m**_ECS_ and ECS filtered local fields. (**a**) The obtained ECS model **m**_ECS_ in black, overlained by a picture of the observed gravity field for reference. The black circle indicates the area, defined in this figure, in which we expect to find ECS representing the local component of the field. Notice that the ECS responsible for the regional field (III) are located in a volume over the edges of the data area. (**b**) ECS filtered local field for western source; (**c**) ECS filtered local field for eastern source. The color bar refers to the gravity field amplitudes.

Many methods have been used to separate interfering potential field anomalies. In the case of a different wavelength content, the separation may consist in the removal of the long wavelength component of a field (the so called “regional field”) from the local effects (the “residual field”). This type of filtering is often referred to as “detrending” and it is usually performed based on a preliminary spectral analysis^12,13^. The simplest detrending techniques are the low-pass filter and the low-order polynomial regression. Problems related to their application are represented by the arbitrary choice of the cutoff wavenumber, the scarce flexibility implied in the assumption of integer orders for the polynomial fitting and the need for performing both filtering and regression considering areas much wider than that of interest. To overcome these issues, other methods have been proposed. For example, Fedi and Quarta (1998) used a decomposition basis, based on the discrete wavelet transform, enjoying a superior space-wavelength resolution and therefore allowing local filtering. Zhang et al.^14^ performed the task using 3-D Principal Component Analysis and textural analysis. A recent approach evaluates the local field by selecting an optimal separation through the computation of fractional-order vertical derivatives (Florio et al.). The ECS filtering procedure was applied to the synthetic gravity field in Fig. 4. To minimize edge errors, the inversion was performed on a volume extending 10 km over the edges of the data area. The obtained ECS model is shown in Fig. 5a. As already said, it consists of equivalent sources, thus they do not expect to represent any realistic geologic body.

**Figure 6:**
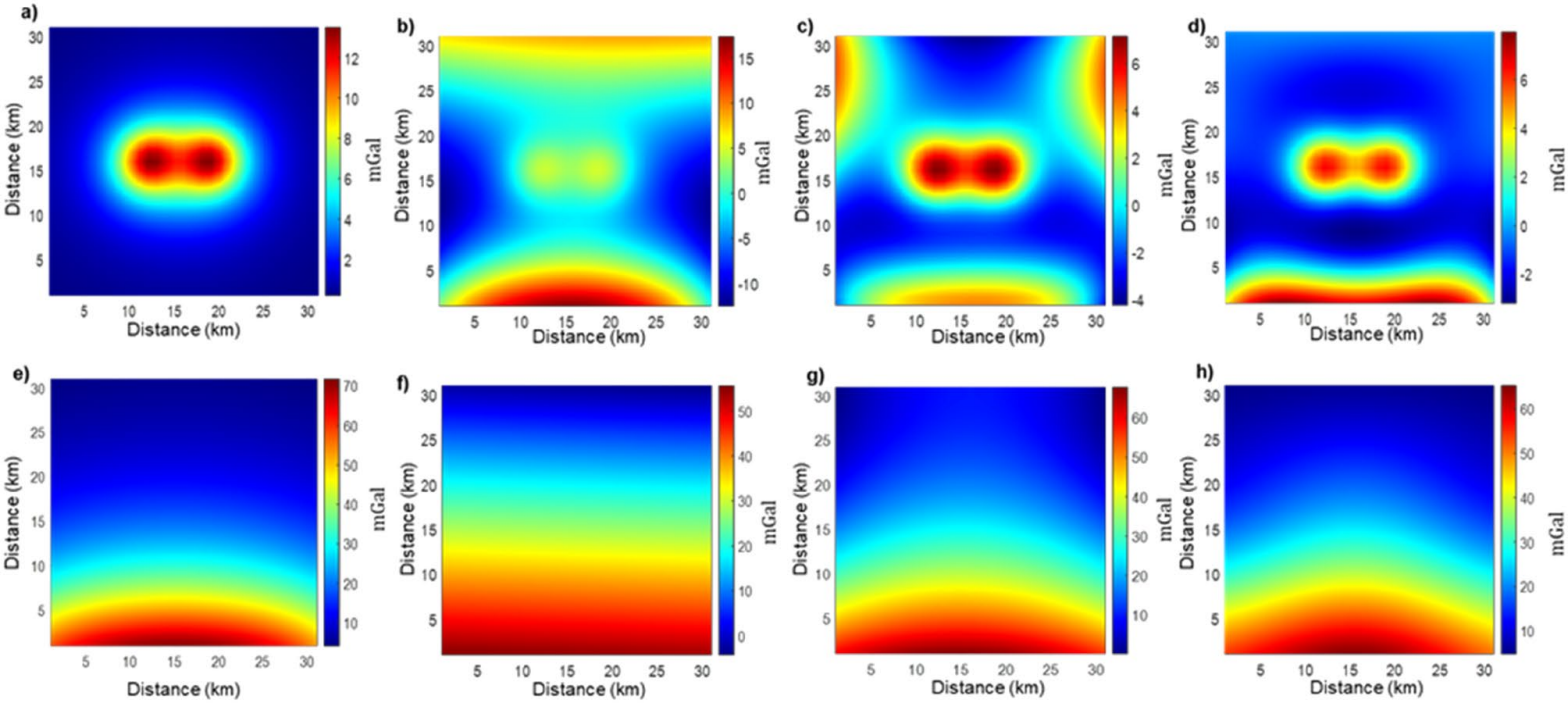
(**a**) Local field estimated with ECS approach; (**b**) Local field estimated by polynomial regression of the first order (**c**) Local field estimated by polynomial regression of the second order; (**d**) Local field estimated by a low-pass filter with cut-off wavelength of 14.2 km. (**e**) Regional field estimated with ECS approach; (**f**) regional field estimated with polynomial regression of the first order; (**g**) regional field estimated with polynomial regression second order; (**h**) regional field estimated with-low pass filter with cut-off wavelength of 14.2 km.

The model in Fig. 5a clearly shows two separated clusters of shallow high-density cells (I and II) that could be interpreted as two distinct local sources, and an elongated group of deep-seated at about 10 km depth dense cells (III) clearly related to the regional field. As the model in Fig. 5a clearly displays two groups of shallow sources, we can isolate from each other the local contributions responsible for the two distinct peaks visible in the maps of Fig. 4b. Thus, we isolated the cells of the group I from those of the group II (Fig. 5) and applied for each model Eq. (9) for obtaining the two local components (Fig. 5b,c).To perform a regional/residual separation, the field generated by the local sources can be retrieved by selecting the shallow ECS in the center of the volume in Fig. 5a and by applying Eq. (9) (Fig. 5b,c). On the other hand, we may obtain the regional field by selecting the deep dense ECS (source III in Fig. 5a) at the edge of the model volume and again applying Eq. (9). The obtained filtered maps of both regional and local fields are shown in Fig. 6a,e. We computed the relative residual as before and found a relative error of 4.5206% for the estimated local field and of 0.53% for the estimated regional field.

We compared our results with those obtainable with common detrending filters (wavenumber domain low pass filtering and polynomial regressions of the first and second order; Fig. 6). The application of the low-pass filter was repeated more times to find the best cut-off wavelength; the best result was found using a cut-off wavelength of 14.2 km. The polynomial regressions and the wavenumber filtering are clearly unable, in this case, to correctly separate the two ensembles of sources. Regional field components are still present in the filtered local component maps (Fig. 6b–d). The local field estimated by polynomial regressions of the first and second order (Fig. 6b,c) show a relative error of 192.37% and 91.19%, respectively, while the retrieved regional field (Fig. 6f–g) show, respectively, relative errors of 22.56% and 10.69%. Local and regional fields estimated by wavenumber filtering (Fig. 6d,h) show, respectively, relative errors of 106.56% and 12.5%.

## Real data application: the local filtering of Mt. Olibano gravity anomaly (Italy)

**Figure 7:**
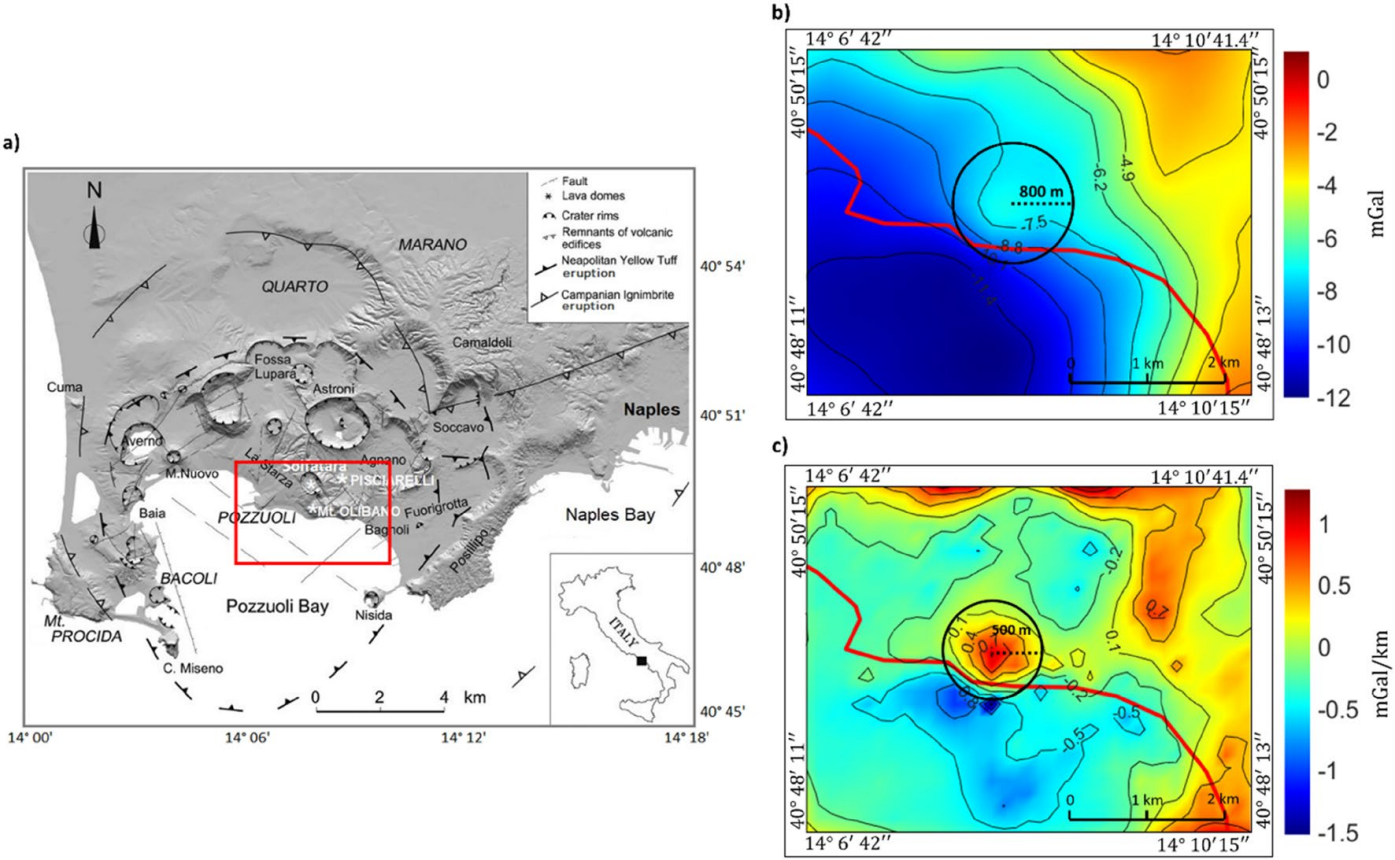
(**a**) Map of the Campi Flegrei caldera (Modified from^18^). The study area is indicated by the red square. Gravity field (**b**) of the study area and its vertical derivative (**c**). The red line (**b**,**c**) indicates the coastline. The black circle (**b**,**c**) indicates the area in which we expect to find dense atoms related to the mount Olibano source.

To demonstrate the utility of the ECS filtering to real data analysis, this method is finally applied to the Bouguer anomalies of the Campi Flegrei volcanic field (near Naples, Southern Italy). The Campi Flegrei (CF) is a volcanic caldera located in the densely populated region encompassing the city of Naples. The volcanic system of CF consists of a dynamic caldera structure that undergo two major collapses during the most significant eruptions of the region (Campanian ignimbrite, approximately 40,000 years ago, and Neapolitan yellow tuff, 15,000 years ago^15–17^). Figure 7a.

**Figure 8:**
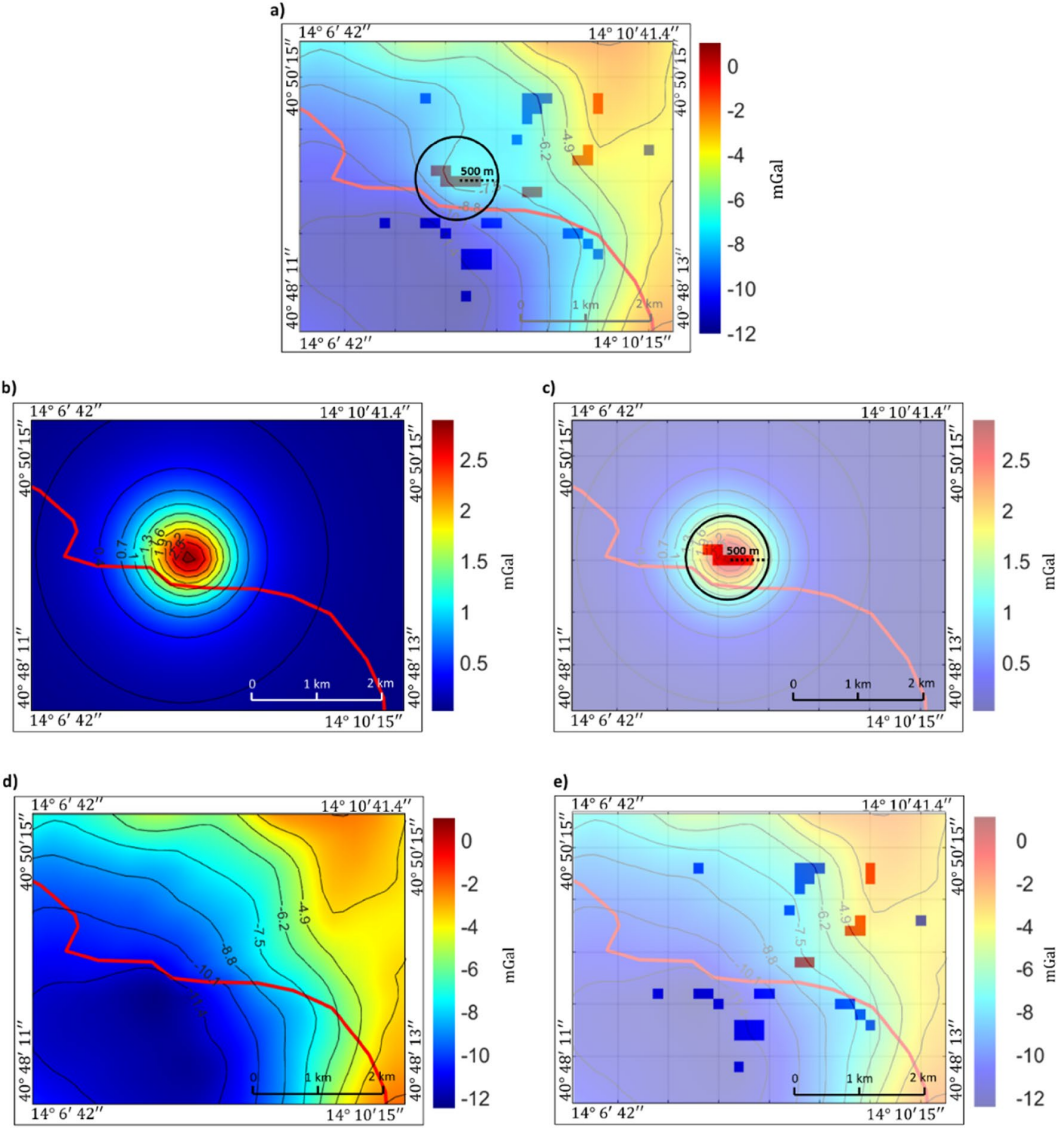
(**a**) ECS model overlained on the gravity field for reference. Positive density atoms are shown as red rectangles, negative atoms are in blue. Notice the presence of the positive density atoms inside the area of interest in the black circle. Is important to note that to obtain a good fitting during the inversion a border in the model is needed in order to adequately fit the regional component of the field. In our case since we are searching for the local component related to Mount Olibano, the source in the border cells are not shown in figure for a better visualization. (**b**) ECS filtered Mt. Olibano lava dome local gravity field; (**c**) Local ECS model (red rectangles represent atoms having positive densities) overlain by the filtered local field in (**b**), (**d**) ECS filtered regional field. (**e**) Regional ECS model (red and blue rectangles represent atoms having positive and negative densities, respectively) overlain by the filtered regional field. In all panels the red line indicates the coastline.

CF area is characterized by a complex morphology, mainly formed by several overlapping volcanic edifices. Most of the volcanic vents are only partially preserved due to both explosive activity and erosion processes. By ECS filtering, we aim at isolating the gravity field generated by a lava dome, located in the presently most active area, from a longer wavelength component generated by the negative density contrast occurring along the caldera ring faults. Figure 7b shows the Bouguer gravity anomalies computed using a 1.4 g/cm^3^ density^19^ in the area relative to the red rectangle in Fig. 7a. This study area includes a general SW decrease of the Bouguer anomalies toward the center of the caldera. A local gravity high, apparently related to the trachytic dome of Mount Olibano, is clearly visible in the central part of the map of Fig. 7b. It is, however, partially masked by the above indicated trend. Our aim is the isolation of the local gravity high from the longer wavelength trend. This would make it possible to have a detailed geophysical modelling of the relative volcanic structure. As a first step, based on the gravity field shape, we define a circular area with a radius of 800 m centered on the target anomaly as that in which we expect to find dense atoms related to the mount Olibano source (black circle in Fig. 7a). However, an analysis of the first vertical derivative (Fig. 7b) indicates that the field of the Mount Olibano is distorted by the presence of the regional trend, and that the source may be better positioned slightly southwest of the gravity high of Fig. 7a. Thanks to the increased resolution of the vertical derivative, we could also narrow to 500 m the radius of the circular area in which we expect to find ECS relative to Mt. Olibano gravity source (Fig. 7b). First, we tried the separation between local and long-wavelength fields using standard approaches (polynomial regressions of the first and second orders and wavenumber filtering), see supporting materials. Then we applied ECS filtering. The obtained ECS model (Fig. 8a) shows several compact sources at different depths. We identify and select a distinct group of dense sources at shallow depths (about 500 m depth) in correspondence to the target area defined in Fig. 7b.

The simple application of Eq. (9) to the selected model cells allowed the computation of the local field (Mt. Olibano anomaly; Fig. 8b), while the residual field, accounting for the regional and other local contributions, can be obtained by the difference between the original and the Mt. Olibano anomaly field (Fig. 8d). The comparison of the residual field (Fig. 8d) with the original gravity data (Fig. 7b) illustrates the good local filtering properties of our method. In fact, the gravity field outside the area interested by the local anomaly is left almost unchanged. The filtering procedure efficiently isolated the gravity contribution of the Mt. Olibano lava dome, showing a gravity anomaly having a single gravity high with a maximum amplitude of about 2.6 mGal. Moreover, the retrieved local anomaly decreases to zero at the edges of the map, demonstrating the absence of any remaining regional field. Comparing our result (Fig. 8b–d) with other methods (Supporting Fig. S1 in Supporting materials) the ECS approach resulted in a more localized filtering, no other contribution seems to be present in the local retrieved field. Moreover, the shape of the regional field looks untouched by the local filtering except from the Mount Olibano gravity field. On the contrary, polynomial regression and wavenumber filtering shows that the wanted local component cannot be completely isolated from other interfering fields as they are characterized by a similar wavenumber content.

## Conclusion

We presented a new method aimed at filtering unwanted contributions by modelling their fields with extremely compact equivalent sources (ECS). Standard use of equivalent sources regards the possibility to compute an equivalent layer^20,21^ to be used for potential field data processing (upward continuation, reduction to the pole, gridding, etc.). These methods are not a significant filtering tool, because tend to yield a continuous distribution of the source property on the selected layer. Contrarily, a local method, such ECS, takes advantage of the extreme source property concentration in the source volume, yielding a sparse source distribution, which may be easily managed for filtering purposes. We want to stress that the progressive compaction of sources of the model (Fig. 2) leading to extremely high source densities and small volumes, allows an unambiguous identification of different interfering contributions.

The main features of our approach are:

- ECS filtering is a form of localized filtering.
- it is possible to separate overlapping field components having similar spectral content.
- it is possible to perform a regional/residual separation.

The inversion is not aimed at recovering a geologically sound model, but it is just a tool yielding an equivalent model, characterized by a distribution of small and well separated sources (ECS sources), whose field still fits adequately the data. About the computational efficiency of our filtering approach, we report the case of our real-data application. The data matrix was of 38 × 27 points and the source volume was subdivided into 48 × 57 × 15 cells, as the model was computed on a larger area to account for edge errors. The inversion process took approximately 1 min on a PC with 1 Intel® Core i7-4790 processor and 32 Gb of RAM. We validated ECS filtering technique on synthetic cases. Even for strongly overlapping anomalies, our technique yielded fairly accurate results, often much better than those obtained with the most common filtering methodologies. The importance and the novelty of our manuscript is the possibility to manage tasks normally impossible with traditional filters, such as the separation of interfering anomalies having a similar wavenumber content. The good filtering properties are also evident by looking at results presented in Fig. 8, relative to the real case application of our filtering approach to the Mount Olibano gravity anomaly (Campi Flegrei volcanic area). Such an accurate localized filtering outperforms the currently available filtering approaches.

In this paper we showed the application of the method to gravity case, but ECS filtering is equally applicable to magnetic data.

## Supplementary Information

Supplementary Information.

## Data Availability

All the data related to synthetic cases can be requested to corresponding author Marco Maiolino. Real case data set is not property of the authors but can be requested to Eni (www.eni.com).
