# Supplementary material for: Extremely compact sources (ECS): a new potential field filtering method
Source: Sci Rep. 2024 May 25;14:11950. doi: 10.1038/s41598-024-62751-3 (PMC11668867; doi:10.1038/s41598-024-62751-3)
Supplement: Supplementary file 1 — Supplementary Information. [file 41598_2024_62751_MOESM1_ESM.docx]

**SUPPORTING MATERIAL**

**Polynomial regression (first and second order) and low pass filtering of the Campi Flegrei Bouguer anomaly**

We show the polynomial regression (first and second order) and low pass filtering of the Campi Flegrei Bouguer anomaly**.** The application of the wavenumber filter was repeated more times, and the best result was found using a 8.5 km cut-off wavelength. The obtained local fields (Supplementary Figure 1 a; b; c) show a prominent gravity high in correspondence to the target anomaly, but, unfortunately, include several others more or less interfering anomalies. The corresponding estimated regional fields are shown in Supplementary Figure 1(d; e; f). The amplitude and the location of the retrieved local anomalies is strictly dependent on the order of the polynomial regression (Supplementary Figure 1 a-b) and the cut-off wavelength (Supplementary Figure 1c). Moreover, it is clear that this kind of filtering is not localized enough to retrieve the amplitude of a single source in complex field. Other contributions are still present in the filtered field (Supplementary Figure 1 a-c), causing interference effects between anomalies that could affect sequent processing steps.


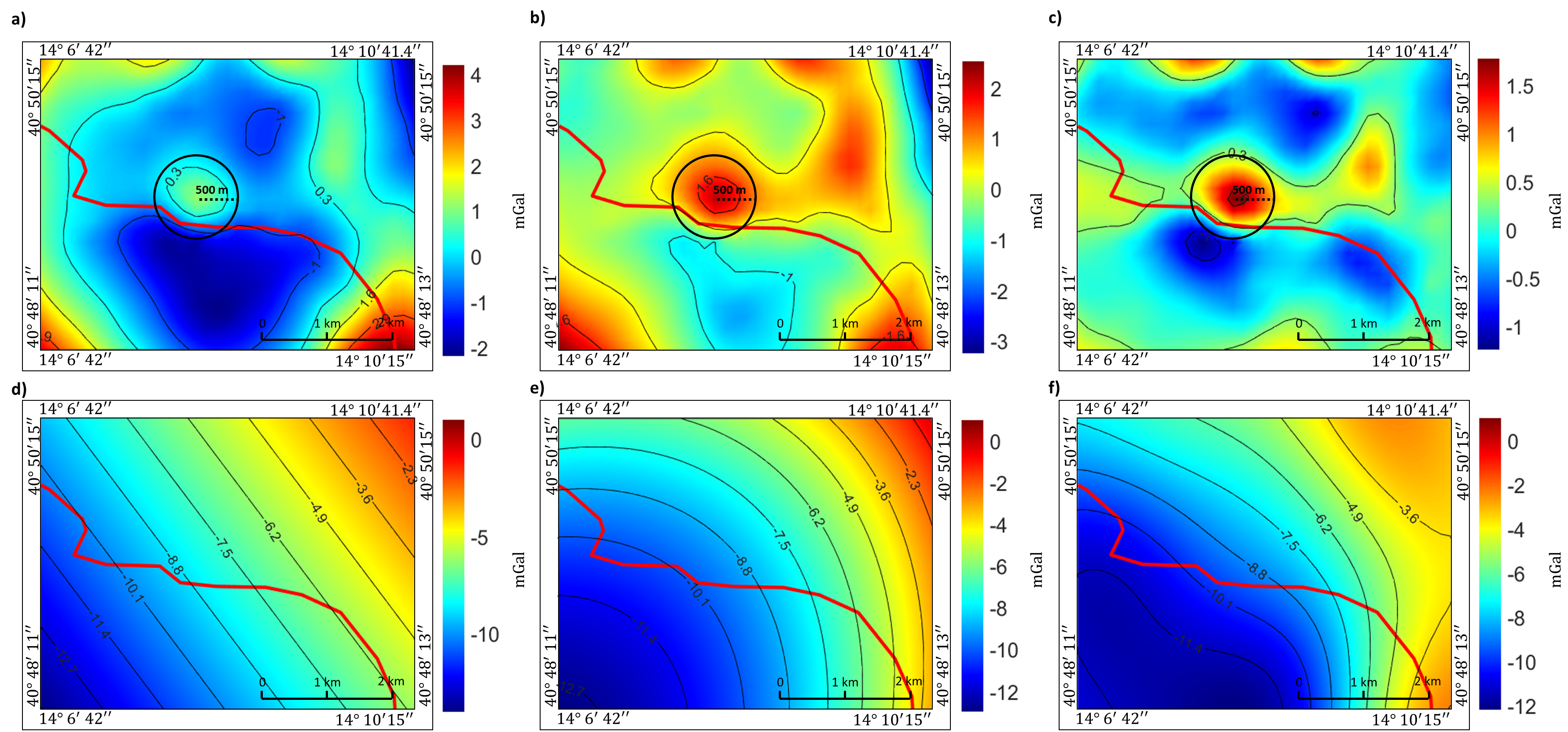


**Supplementary Figure 1.** Regional and local fields estimated by using standard approaches. a) Residual gravity field estimated by polynomial regression of the first order. b) Residual gravity field estimated by polynomial regression of the second order. c) Residual gravity field estimated by a low-pass filter with cut-off wavelength of 8.5 km. d) Regional gravity field estimated by polynomial regression of the first order. e) regional gravity field estimated by polynomial regression of the second order. f) regional gravity field estimated by a low-pass filter with cut-off wavelength of 8.5 km. The red line indicates the coastline. The black circles in panels a), b) and c) indicate the area in which we expect to find ECS relative to Mt. Olibano gravity anomaly source. Maps a-f have been created using Matlab R2023b (https://it.mathworks.com/products/new_products/release2023b.html) with property codes.
